# EPI-Trans: an effective transformer-based deep learning model for enhancer promoter interaction prediction

**DOI:** 10.1186/s12859-024-05784-9

**Published:** 2024-06-18

**Authors:** Fatma S. Ahmed, Saleh Aly, Xiangrong Liu

**Affiliations:** 1 Department of Computer Science and Technology, Xiamen University, Xiamen, 361005 China; 2 Department of Electrical Engineering, Aswan University, Aswan, 81542 Egypt; 3 Department of Information Technology, Majmaah University, 11952 Majmaah, Saudi Arabia

**Keywords:** Enhancer, Enhancer–promoter interaction (EPI) prediction, Promoter, Transformer

## Abstract

**Background:**

Recognition of enhancer–promoter Interactions (EPIs) is crucial for human development. EPIs in the genome play a key role in regulating transcription. However, experimental approaches for classifying EPIs are too expensive in terms of effort, time, and resources. Therefore, more and more studies are being done on developing computational techniques, particularly using deep learning and other machine learning techniques, to address such problems. Unfortunately, the majority of current computational methods are based on convolutional neural networks, recurrent neural networks, or a combination of them, which don’t take into consideration contextual details and the long-range interactions between the enhancer and promoter sequences. A new transformer-based model called EPI-Trans is presented in this study to overcome the aforementioned limitations. The multi-head attention mechanism in the transformer model automatically learns features that represent the long interrelationships between enhancer and promoter sequences. Furthermore, a generic model is created with transferability that can be utilized as a pre-trained model for various cell lines. Moreover, the parameters of the generic model are fine-tuned using a particular cell line dataset to improve performance.

**Results:**

Based on the results obtained from six benchmark cell lines, the average AUROC for the specific, generic, and best models is 94.2%, 95%, and 95.7%, while the average AUPR is 80.5%, 66.1%, and 79.6% respectively.

**Conclusions:**

This study proposed a transformer-based deep learning model for EPI prediction. The comparative results on certain cell lines show that EPI-Trans outperforms other cutting-edge techniques and can provide superior performance on the challenge of recognizing EPI.

**Supplementary Information:**

The online version contains supplementary material available at 10.1186/s12859-024-05784-9.

## Background

Enhancers, promoters, and other regulatory elements in non-coding genomic regions play important roles in transcriptional control. The enhancer and promoter interactions, in particular, regulate gene expression in a coordinated way. Although enhancers and promoters may be physically separated in the genome, they can be closely associated and connected by chromatin looping in a 3D space. Some enhancers also interact with the target promoters by avoiding communicating with neighboring promoters in response to histone or transcriptional genomic changes. A precise mapping of such remote connections is of particular relevance for comprehending gene expression pathways and determining target genes of genome-wide association studies(GWAS) loci [1–3]. Experiments that capture chromosomal conformation (3C, 4C, and Hi-C) or extend ChIP-sequencing techniques like paired-end tag sequencing (ChIA-PET) are costly and only provide results for a small number of cell types [4–7]. An alternative is provided by computational techniques that predict EPIs using machine learning models based on empirically acquired EPI data utilizing distinct DNA sequence and/or epigenomic annotation data [8–11].

In many pattern recognition tasks, neural networks have been successfully applied [12], and deep learning has become a common method for building predictive models based on DNA sequences [12–16], and other bioinformatics studies [17, 18]. The advantage of the deep learning framework is that it can predict certain functional annotations by automatically extracting valuable features from the genome sequence and identifying nonlinear correlations in the sequence [19]. Mostly, EPI identification and detection are carried out either by wet experiments in the laboratory or by various data mining techniques. Wet experiments require complex designs and require much time to perform. Therefore, they are inefficient for EPI screening.

In recent years, several computational techniques based on machine learning have been presented and shown to be effective in quickly and efficiently identifying EPIs. These techniques may be broadly categorized into two groups: the first group is based on genomic data and the second one is based on sequence. In the techniques of the first set, classifiers are trained using characteristics extracted from genomic data to discriminate between EPIs. For instance, Whalen et al. [11] introduced TargetFinder, a model was trained with different genomic data to predict EPIs, including transcription factor ChIP-seq, histone marks, DNA methylation, DNase-seq, CAGE, and gene expression data. However, since it needs a specific understanding of how to choose genetic features, this type of method is constrained. The second group relies solely on information from sequences to identify EPIs. For example, Yang et al. [20] introduced a prediction technique that trained a model of a boosted tree ensemble to derive features directly from genomic sequences using word embedding. The identification of EPIs by an attention-based neural network model, known as EPIANN, was also pioneered by Mao et al. [21]. EPIANN incorporates a location-based feature decoding algorithm and an attention mechanism to enhance performance. Singh et al. [22] introduced the SPEID, a prediction model based on deep learning, which combines long short-term memory (LSTM) with the convolutional neural network (CNN). Zhuang et al. [23] simplified the SPEID model and constructed a predictive model SIMCNN, that uses CNN in conjunction with transfer learning to train its model.

Several methods have been developed that combine CNN with recurrent neural networks to predict enhancer–promoter interactions (EPIs) solely based on DNA sequence information. Hong et al. [24] presented EPIVAN, which encodes enhancers and promoters using DNA vectors pre-trained with whole human genome sequences. They then extracted local and global characteristics using a 1D convolution network and gated recurrent units, and they used the attention mechanism to increase the contribution of key features. Min et al. [25] proposed EPI-DLMH, a model that utilizes a two-layer convolutional neural network (CNN) and a bidirectional network with gated recurrent unit (GRU) to extract local and long-range dependencies from promoter and enhancer sequences. An attention mechanism is then employed to focus on the most significant features, and a matched heuristic mechanism is used to analyze the relationship between promoters and enhancers. Furthermore, Wang et al. [26] developed EPnet, a deep learning model that uses a combination of CNN and bidirectional GRU to extract important features from the DNA sequences. The performance of the model as a whole is enhanced by the output module’s subsequent use of a CNN and dense layer combination to further enhance these important properties. Recently, Fan and Peng [27] introduced a technique known as StackEPI, which merges several feature representations and classical machine learning algorithms, employs a stacking ensemble approach, and performs the prediction process solely based on promoter and enhancer gene sequences.

The majority of the aforementioned approaches use Convolution Neural Network (CNN)-based architecture [28], and other tools like Long Short-Term Memory (LSTM) [29] and Gated Recurrent Units (GRU) [30]. Recurrent neural network (RNN)-based models capture the dependency between states to focus on the sequential properties of DNA. Some hybrid strategies were also developed to combine the benefits of the two model designs [31–33]. For a better EPI model, an optimal computational approach should take into account all contextual details to extract efficient features from sequences. However, neither the CNN nor the RNN architectures can meet these demands [34, 35]. Since CNN’s capacity to extract local characteristics is limited by filter size, it often fails to grasp semantic dependency in long-range settings. While RNN (LSTM, GRU) models are capable of learning long-term dependency, they are severely hindered by gradient and low-efficiency issues since they process all prior states sequentially and condense contextual information into a bottleneck of lengthy input sequences. To address the drawbacks described above, the transformer mechanism [36] is utilized by Yu et al. [37] to build a new model called EPI-mind. Transformer is an attention-based architecture that draws global dependencies between input and output and has attained cutting-edge effectiveness in most natural language processing tasks. Although EPI-mind achieved good performance, there is still room to do more improvement. Where EPI-mind used two transformers, one for the enhancer sequence and another one for the promoter sequence, then combined the output features of the two transformers. Since the main purpose of the transformer is to handle one sequence and extract the relationship between the words or tokens for DNA sequence, we proposed a new model called EPI-Trans which first combines the output feature vectors from the convolution layers of the enhancer and promoter then fed these merged features to the transformer module as a single sequence. The transformer in this case jointly extracts the features of the enhancer and the promoter and hence learns the relationship between them more accurately. The Query, Key, and Value matrices used as input to the multi-head attention is the combination of the enhancer and promoter features. In addition, using a single transformer module and a single encoder inside the transformer reduces the computation complexity and speeds up the training of the model, thus our model is less complicated, more accurate, and provides higher performance.

## Methods

### Data

In this study, we compared our model with previous approaches using the same TargetFinder EPIs dataset [11]. The data comprises enhancer/promoter sequences from six human ENCODE cell lines: K562 (mesoderm-lineage cells derived from a patient with leukemia), GM12878 (lymphoblastoid cells), HeLa-S3 (ectoderm-lineage cells derived from a patient with cervical cancer), HUVEC (umbilical vein endothelial cells), IMR90 (fetal lung fibroblasts), and NHEK (epidermal keratinocytes). Whalen et al. [11] detected active promoters and enhancers in each cell line by utilizing segmentation-based annotations from ENCODE and Roadmap Epigenomics, along with gene expression data from ENCODE. The researchers classified all enhancer–promoter pairs as either interacting or non-interacting, using high-resolution genome-wide measurements of chromatin contacts in each cell line. Interacting pairs were considered as positive examples, while non-interacting pairs were considered as negative examples. A significant number of these pairs were also identified using capture Hi-C. A sample of non-interacting pairs was taken, with 20 pairs per interacting pair, to match the enhancer–promoter distances of the interacting pairs. All distances were less than 2 Mb. They constructed feature lists for all enhancer–promoter pairings in each cell line by utilizing functional genomics data, including metrics for open chromatin, DNA methylation, gene expression, and ChIP-seq peaks for transcription factors, architectural proteins, and modified histones. The signal was measured at the promoter, enhancer, and at the genomic region between them. In addition, they calculated characteristics for the preserved arrangement of the enhancer and promoter, as well as the resemblance between the annotations of transcription factors and target genes, which are linked to interactions that have been empirically confirmed.

The length of the enhancer and promoter sequences is 3000 bp and 2000 bp respectively. Each cell line has a 1:20 ratio of positive to negative examples, with 20 negative instances chosen for every positive example. Using an imbalanced dataset for training in a supervised deep learning model would result in an excessive emphasis on the predominant class, leading to a decreased accuracy of the minority class and a negative model bias prediction. To solve this problem, we employed the same data augmentation technique used in [21] to balance the classes by amplifying the training set’s positive samples 20 times. This was achieved by sliding a window with a fixed size from the right or left over the DNA sequences while ensuring that the extended region still contains most of the functional parts. The result was a balanced dataset, as shown in Table 1.

**Table 1:** Number of positive samples, augmented positive samples, and negative samples for each cell line in the training and testing datasets

Cell Lines	Training Dataset			Test Dataset	
	Pos Samples	Aug. Pos Samples	Neg Samples	Pos Samples	Neg Samples
GM12878	1902	38040	37980	211	4220
HeLa-S3	1566	31320	31320	174	3480
HUVEC	1372	27440	27360	152	3040
IMR90	1129	22580	22500	125	2500
K562	1780	35600	35550	197	3950
NHEK	1162	23240	23040	129	2560
Total	-	178220	177750	-	-

### Model structure

**Fig. 1:**
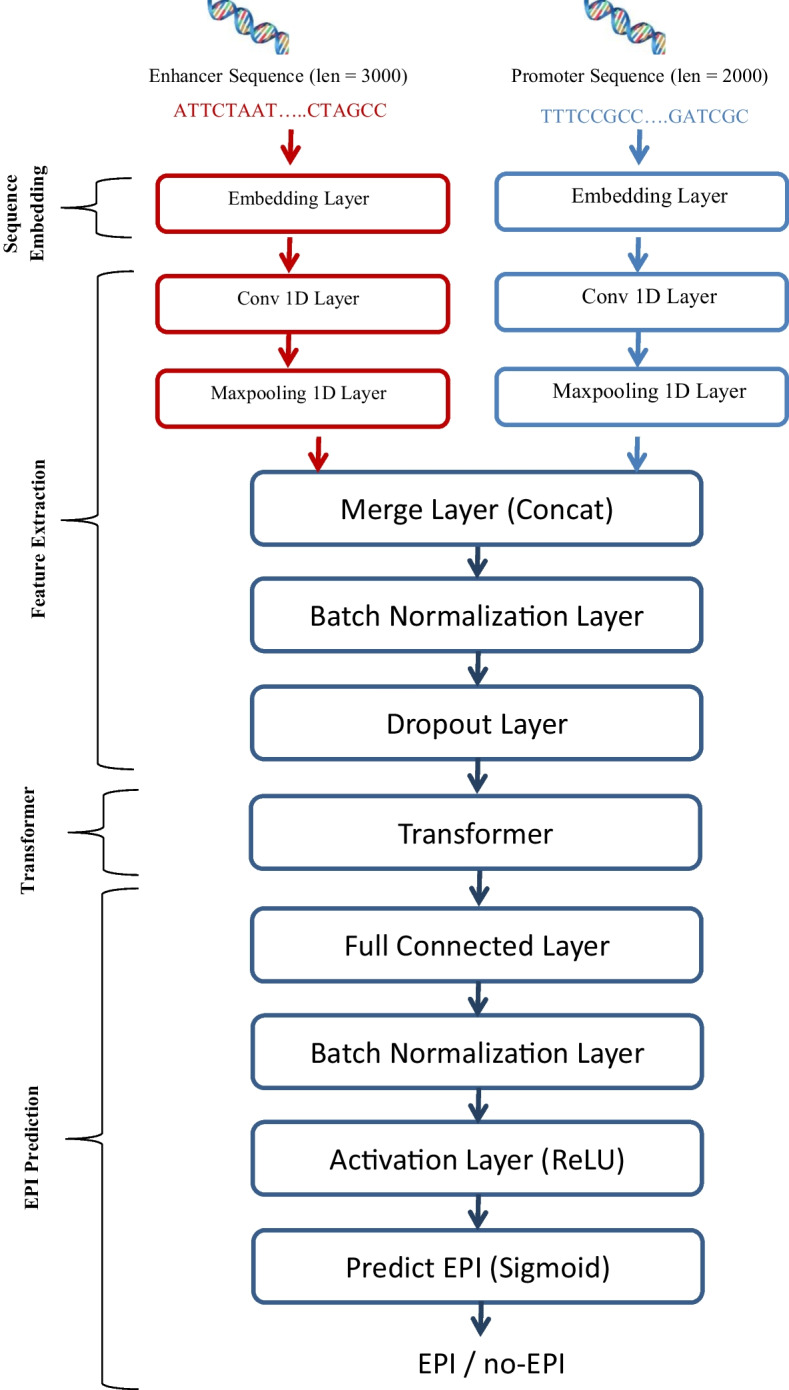
Structure of the proposed EPI-Trans Model which consists of sequence embedding, features extraction, transformer, and finally prediction of EPI

We propose a transformer-based approach for the automatic detection of EPIs using DNA sequences. Figure 1 illustrates the proposed predictive framework, which consists of four key steps: sequence embedding, feature extraction, transformer, and EPI prediction. Firstly, enhancer and promoter sequences are fed into the model as input and embedded as feature matrices using the dna2vec embedding method. Then, a hybrid multilayer convolutional neural network is employed to learn high-level features from these feature matrices. These features are subsequently passed through the transformer module and then the prediction layer to determine the existence of an interaction between enhancers and promoters. We present the proposed framework in detail in the following.

#### Sequence embedding

In this study, we used the k-mer representation method to analyze long DNA sequences. Following the representation of the k-mer, promoters and enhancers were separated using a k-bp window with a sliding step size of *s*. Previous studies have shown that setting *k* to 6 yields optimal results for computational effectiveness and information complexity of the vectors [20]. Thus, we set *k* and *s* to 6 and 1 respectively. For example, the sequence ‘ACGGTTTA’ was divided into ‘ACGGTT’, ‘CGGTTT’, and ‘GGTTTA’ using k-mer representation. There are two methods to embed the DNA sequences, dna2vec and one-hot embedding methods. Although one-hot vector encoding is a simple and easy-to-compute method, it is susceptible to the curse of dimensionality problem. The dimension of the one-hot vector is specifically exponential to the length of *k*. For example, a 6-mer needs a bit vector with a $4^6$ (4096)-dimensional size. Because the majority of deep learning algorithms prefer lower-dimensional continuous vectors as input, this presents a challenge in biological sequence analysis [19].

**Fig. 2:**
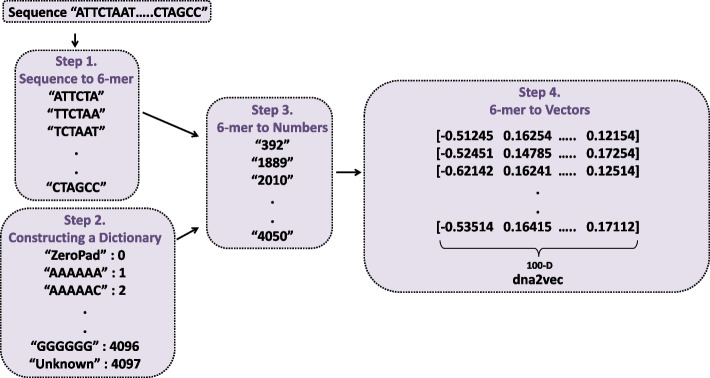
Process of enhancer/promoter sequence embedding using dna2vec embedding method

To address these issues, we utilized the dna2vec embedding method [38, 39]. Dna2vec embedding is based on the word2vec model [40], which produces low-dimensional vectors of high quality to represent k-mer words. The dna2vec approach introduced an innovative technique for computing distributed representations of k-mers with varying lengths. These k-mers exhibit consistency across various lengths, meaning that they are inside the same embedding vector space. The algorithm maps k-mers of length 3–8, where 3 is the minimum length and 8 is the maximum length, into a vector space with 100 dimensions. The model employed a shallow neural network with two layers to train a collective DNA k-mer embedding. The model was trained using the hg38 human assembly from chromosome 1 to chromosome 22 [41]. Thus, we used dna2vec to represent the enhancer/promoter sequences with 6-mer tokens, resulting in a $3000 \times 100-D$ matrix for the enhancer sequences and a $2000 \times 100-D$ matrix for the promoter sequences. Figure 2 shows the embedding process of the enhancer/promoter sequences using the dna2vec embedding method (please note that this is an example, and the actual length of the enhancer and promoter sequence is 3000bp and 2000bp respectively).

#### Feature extraction

We utilized a 2-layer CNN network to process input from the promoter and enhancer sequences. Specifically, we employed two separate CNNs: one dedicated to the enhancer and the other to the promoter. Each CNN consisted of a single 1D convolution layer followed by a single max-pooling layer. Learning local features from enhancer and promoter input is achieved through the 1D convolution layer, with the subsequent max-pooling layer serving to reduce feature dimensions. Following the convolution operation, an activation layer utilizing the ReLU function is applied. The model captures distinct features for both the enhancer and promoter sequences, and these features are then concatenated using a merge layer. To mitigate overfitting, batch normalization and dropout layers have been incorporated into the model after the merge layer.

#### Transformer

**Fig. 3:**
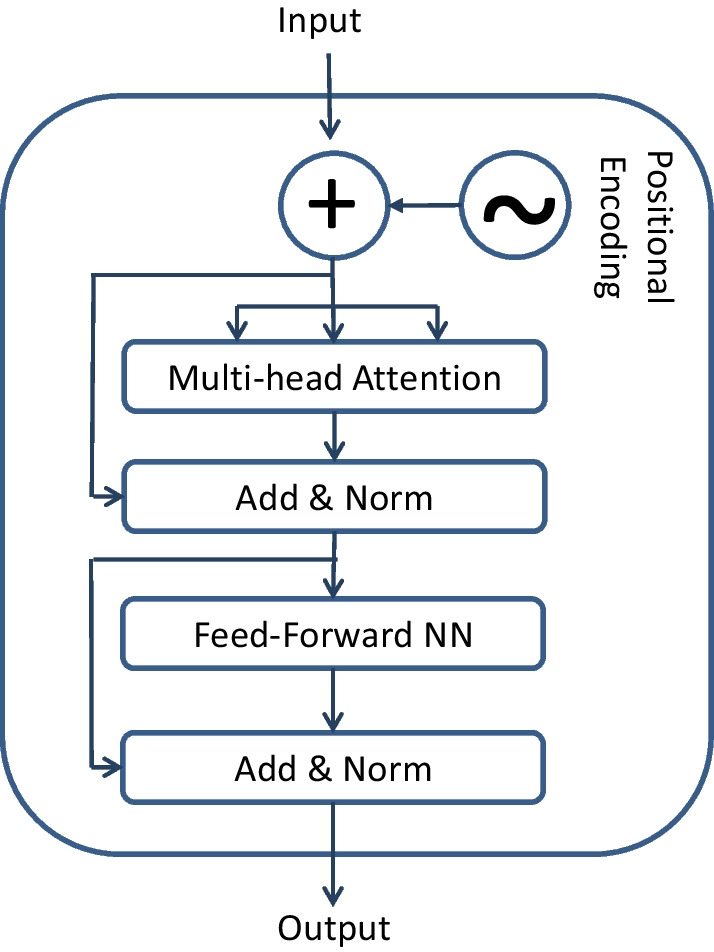
Structure of the transformer module

We employed the transformer technique, initially proposed by Vaswani et al. [36], to extract high-level or global features. Due to the transformer mechanism’s inherent ability to capture positional information, it can automatically acquire additional features. Figure 3 illustrates the transformer mechanism, comprised of four modules: positional encoding, multiple-head attention, position-wise feedforward network, and add &norm. Vaswani et al.’s work provides a detailed explanation of the transformer mechanism.

There are some constraints that govern the hyperparameters of both the 2-layer CNN and the transformer. Firstly, the number of filters in the 1D convolutional layer is tied to the model dimension of the transformer. The add &norm layer, positioned at the beginning of the transformer, combines the input of the transformer with the output of the multi-attention heads. Consequently, the number of filters must match the model dimension. Secondly, within the transformer, since the model dimension is divided among the multi-head attentions, it is imperative that the model dimension be a multiple of the number of attention heads.

Numerous experiments have been conducted to validate the hyperparameters, taking into account the specified constraints for both the transformer and the CNN. The hyperparameter values yielding the best performance have been selected based on these experiments. Consequently, the filter size for the 1D convolution layer in the enhancer and promoter is set to 80 and 61, respectively, with a stride of 1 for both. The 1D max-pooling layer has a pool size of 15 and 10 for the enhancer and promoter, respectively, with the stride size being equal to the pool size. Both the model dimension of the transformer and the number of filters in the 1D convolution layer are set to 72. The transformer module is configured with 1 encoder stack, 9 multi-head attentions, and 256 hidden units for the feedforward layer.

#### Sequence prediction

The final step in the process entails forwarding the generated feature vector to a fully connected layer (dense layer) comprising 50 neurons to generalize from these features into the output space. To prevent overfitting, where a network memorizes training instances and noise rather than capturing the underlying relationship, our model needs regularization. The standardizing and normalizing processes on the layer input from the dense layer are carried out via batch normalization, as a result, the network is prevented from becoming dependent on a certain subset of inputs. Then the output of the batch normalization is passed to ReLU activation function. Finally, the feature vector is passed through a single unit with a sigmoid activation function to produce the final output. The resulting probability score indicates the likelihood that the input sequences will result in an interaction between the enhancer and promoter.

### Model training and testing

The interaction between the enhancer and the promoter is determined by the specificity of the cell line. Different cell lines have different rules for this interaction. Hence, a model constructed from one cell line may not be transferable to another. We train and test a model separately for each cell line. To assess the performance of our proposed model compared to existing models, we used identical training and test sets for each cell line as employed in previous works. The subsequent procedures outline the training methodology employed for each cell line.

1. The imbalanced dataset *D* was split into a training set (90% of *D*) and a test set (10% of *D*) using stratified sampling to ensure that the class distribution was preserved in both sets.
2. To address the class imbalance in the training set, the minority class was oversampled as mentioned above in the data section, resulting in a balanced dataset ($D_{aug}$).
3. The balanced training dataset $D_{aug}$ was split into training set $D_{train}$ (95% of $D_{aug}$) and validation set $D_{val}$ (5% of $D_{aug}$).
4. The proposed model was trained on the training set $D_{train}$, and validated on the validation set $D_{val}$ for a suitable optimization algorithm and hyperparameters tuning.
5. The model was tested in the test set $D_{test}$ using the standard evaluation metrics area under the receiver operating characteristic curve (AUROC) and the area under the precision–recall curve (AUPR).

Numerous experiments were conducted using various optimizers [42], including Nesterov-accelerated adaptive moment estimation (Nadam) [43], Stochastic Gradient Descent (SGD), Adaptive Moment Estimation (Adam), RMSprop, and Adamax. Different values for the learning rate (0.01, 0.001, and 0.0001) and batch size (16, 32, 64, 128, and 256) were employed during model training. The experiments were executed for 15, 20, 25, and 30 epochs. Additional file 1: Tables S1 and S2, S3 and S4, S5 and S6, and S7 and S8 present the results obtained with different optimizers, learning rates, batch sizes, and epochs, respectively, in terms of AUROC and AUPR in the supplementary materials.

In light of the conducted experiments, the ultimate values for the hyperparameters were chosen based on the superior average performance of AUROC and AUPR across the six cell lines. Therefore, the Nadam optimizer was employed to minimize the loss with a learning rate of 0.001. The model used a mini-batch size of 64 samples during backpropagation with binary cross-entropy loss, and the number of epochs was set to 20. The model was trained on a server with a GeForce GTX 2080 Ti GPU with 11GB RAM and a total memory of 251 GB. The server runs Ubuntu 18.04 LTS, and the software installed includes cuda 10.2, conda 4.7.10, Python 3.7, and the versions of the other used software libraries and frameworks mentioned in a text file called “requirements.txt” at the GitHub repository.

### Evaluation metrics

The assessment criteria employed in this study were area under the precision–recall curve (AUPR) [44] and area under the receiver operating characteristic curve (AUROC) [45], which allowed for comparison with state-of-the-art methods. ROC is a curve that plots the sensitivity (TPR) against specificity (FPR) at various threshold values. In other words, it shows the performance of a classification model at all classification thresholds. The area under the ROC curve region is known as AUROC. The model’s performance improves as the AUROC value approaches 1 and the curve approaches the top left corner. Because the ROC curve is unaffected by the distribution of positive and negative data, the AUROC is an effective assessment metric for the model used for imbalanced data. The precision–recall curve represents the trade-off between the precision of the model’s detection of positive examples and the model’s capacity to cover positive cases, with precision as the vertical axis and recall as the horizontal axis. The AUPR is the area under the precision–recall curve. The model’s performance improves as the AUPR value approaches 1 (or as the curve approaches the upper right corner).

## Results and discussion

### Performance of cell line specific model

The model that uses this specific training methodology is referred to as EPI-Trans-specific. For cross-cell line evaluation, the AUROC and AUPR of EPI-Trans-specific are displayed in Tables 2 and 3, respectively. Results reveal that EPI-Trans-specific performs well at predicting EPIs when the sets used for training and testing are from the same cell line. In the cross-cell line test, the model performed significantly worse compared to its performance when trained and tested on the same cell line. When employing the same cell line for training and testing the EPI-Trans-specific model, the performance in terms of AUROC is 0.938, 0.963, 0.939, 0.898, 0.931, and 0.984, and in terms of AUPR is 0.797, 0.854, 0.736, 0.733, 0.783, and 0.927 for cell lines GM12878, HeLa-S3, HUVEC, IMR90, K562, and NHEK, respectively. The results suggest that predicting enhancer–promoter interactions (EPIs) on other cell lines based solely on the sequence perspective of a specific cell line is not accurate. The model trained on a particular cell line can only learn the interaction patterns between enhancers and promoters specific to that cell line. Conversely, this implies that enhancer–promoter interactions are cell-line specific.

**Table 2:** Performance of EPI-Trans-specific model using AUROC performance index on six cell lines

Train/Test cell lines	GM12878	HeLa-S3	HUVEC	IMR90	K562	NHEK
GM12878	**0.938**	0.683	0.668	0.647	0.643	0.590
HeLa-S3	0.598	**0.963**	0.645	0.584	0.593	0.603
HUVEC	0.627	0.700	**0.939**	0.643	0.626	0.613
IMR90	0.629	0.581	0.614	**0.898**	0.610	0.619
K562	0.616	0.641	0.655	0.630	**0.931**	0.640
NHEK	0.586	0.580	0.655	0.558	0.629	**0.984**

The best performance in each cell line is given in boldface

**Table 3:** Performance of EPI-Trans-specific model using AUPR performance index on six cell lines

Train/Test cell lines	GM12878	HeLa-S3	HUVEC	IMR90	K562	NHEK
GM12878	**0.797**	0.151	0.171	0.165	0.128	0.094
HeLa-S3	0.098	**0.854**	0.235	0.105	0.109	0.174
HUVEC	0.098	0.233	**0.736**	0.132	0.169	0.171
IMR90	0.106	0.097	0.117	**0.733**	0.106	0.127
K562	0.140	0.161	0.170	0.126	**0.783**	0.143
NHEK	0.109	0.126	0.156	0.115	0.122	**0.927**

The best performance in each cell line is given in boldface

### Performance of generic model trained on all cell lines

In the previous experiments, training six distinct EPI-Trans-specific models, one for each cell line proved to be time-consuming. The second generic strategy is more effective, involving the training of a single model using collective data from the six cell lines and is more akin to traditional transfer learning [46]. We have developed a generic model, EPI-Trans-generic by training a single model using combined dataset from all six cell lines. This model can predict the EPIs for any cell line used in the training.

We hypothesized that enhancer–promoter interactions (EPIs) might exhibit certain shared features across different cell lines, in addition to the cell line-specific features. The generic model is effective in capturing common features among cell lines, particularly when the training set includes sufficiently distinct cell lines. According to this hypothesis, a new training set, D_All_ is produced by combining and disrupting the training sets of the six cell lines. D_All_ includes all the enhancer–promoter pairs of the augmented/balanced datasets of the six cell lines, and the ratio for enhancer–promoter pairs is 1:1:1.2:1.4:1.6:1.7 of the six cell lines IMR90:NHEK:HUVEC:HeLa-S3:K562:GM12878, respectively, so it contains almost close ratio from all six cell lines. D_All_ is considered a balanced dataset where the number of negative samples is 177,750 and the number of positive samples is 178,220 as shown in Table 1.

The generic model is trained using D_All_ dataset for 20, 25, and 30 epochs, followed by separate evaluations on each specific cell line test set. Additional file 1: Tables S9 and S10 in the supplementary materials present the performance results for various epoch numbers in terms of AUROC and AUPR, respectively. The generic model is trained for 25 epochs, as it achieved the best average performance in terms of both AUROC and AUPR. This model demonstrates performance with AUROC results of 0.944, 0.963, 0.944, 0.933, 0.942, and 0.975 for cell lines GM12878, HeLa-S3, HUVEC, IMR90, K562, and NHEK, respectively. Correspondingly, AUPR results are 0.643, 0.749, 0.584, 0.611, 0.658, and 0.723 for the mentioned cell lines, as indicated in Table 4. On the contrary, upon comparing the results of the EPI-trans-generic model with those of the EPI-Trans-specific model in terms of AUPR, as depicted in Table 6, it becomes evident that the performance of the EPI-trans-generic model is inferior to that of the EPI-Trans-specific model for all the cell lines. This observation aligns with the earlier discussion that emphasized the dependence of enhancer–promoter interactions (EPIs) on specificity features within a specific cell line. While the EPI-Trans-generic model excels in capturing common features, it is less adept at capturing particular features compared to the EPI-Trans-specific model. Despite this, the EPI-Trans-generic model remains a robust generic model for predicting EPIs across the diverse set of six cell lines.

**Table 4:** Performance of EPI-Trans-generic model in the index of AUROC and AUPR on six cell lines

Cell lines	GM12878	HeLa-S3	HUVEC	IMR90	K562	NHEK
AUROC	0.944	0.963	0.944	0.933	0.942	0.975
AUPR	0.643	0.749	0.584	0.611	0.658	0.723

### Performance of the EPI-Trans-best model that used EPI-Trans-generic model as a pre-trained model

From the results of the EPI-Trans-generic model, it is evident that cell line-common features were successfully captured while cell line-specific features could not be effectively captured. Despite its adaptability across the six cell lines, the performance of the EPI-Trans-generic model was somewhat inferior to that of the EPI-Trans-specific model. Therefore, to enhance the performance of the generic model, we applied an alternative training method called the “best training method” allowing the generic model to learn specific cell line features. The model trained using this training strategy is referred to as EPI-Trans-best. The process of training is defined as follows:

1. Using the EPI-Trans-generic model mentioned in the previous section as a pre-trained model.
2. Fine-tuning the parameters of the generic model by training it on the training set of a particular cell line for *n* epochs (where *n* = 20, 25, and 30). Additional file 1: Tables S11 and S12 in the supplementary materials show the detailed results due to using different epochs in terms of AUROC and AUPR respectively. The number of epochs is selected to be 30 for the best model because the average AUROC and AUPR are the best when using this value.
3. Using the testing set of that particular cell line to evaluate the new model.

The performance of the new model was assessed after applying the best training strategy. Tables 5 and 6 shows the results of the EPI-Trans-best model in terms of AUROC and AUPR respectively using each of the six cell lines. The best model achieves higher performance than the EPI-Trans-specific model in five cell lines in terms of AUROC, where the performance reaches 0.946, 0.964, 0.952, 0.941, and 0.956 for cell lines GM12878, HeLa-S3, HUVEC, IMR90, and K562 respectively. The new training strategy increases the performance of EPI-Trans-specific by 0.8%, 0.1%, 1.3%, 4.3%, and 2.5% respectively. On the other hand, when comparing the performance of the best model with the generic model, an improvement is observed in all cell lines for both metrics AUROC and AUPR. This demonstrates that the best model effectively learns from the generic model.

**Table 5:** Comparison between the three models of EPI-Trans in the index of AUROC

Model/Cell lines	GM12878	HeLa-S3	HUVEC	IMR90	K562	NHEK
EPI-Trans-spec	0.938	0.963	0.939	0.898	0.931	**0.984**
EPI-Trans-gen	0.944	0.963	0.944	0.933	0.942	0.975
EPI-Trans-best	**0.946**	**0.964**	**0.952**	**0.941**	**0.956**	0.983

The best performance in each cell line is given in boldface

**Table 6:** Comparison between the three models of EPI-Trans in the index of AUPR

Model/Cell lines	GM12878	HeLa-S3	HUVEC	IMR90	K562	NHEK
EPI-Trans-spec	**0.797**	0.854	**0.736**	0.733	**0.783**	**0.927**
EPI-Trans-gen	0.643	0.749	0.584	0.611	0.658	0.723
EPI-Trans-best	0.778	**0.857**	0.724	**0.758**	0.758	0.901

The best performance in each cell line is given in boldface

### Computational complexity of the proposed EPI-Trans models

In this section, we provide insights into the computational aspects of our proposed EPI-Trans models. The training and test times for each cell line are detailed in Additional file 1: Tables S13 and S14. Table 7 consolidates the average training and test times, offering a comprehensive view across all samples for the six cell lines. A comparative analysis of the average training duration for all samples reveals that the EPI-Trans-general model requires more time (5.3 h). This extended duration is attributed to the use of a larger number of samples in training, incorporating data from all cell lines. On the contrary, the average testing time for all three models remains nearly identical, as they undergo evaluation on the same dataset with an equal number of samples for each cell line.

**Table 7:** The average training and test time of EPI-Trans models for all samples and per a sample respectively

Model	Avg Training Time All Samples (hour)	Avg Testing Time Per a Sample (msec)
EPI-Trans-spec	0.849	0.504
EPI-Trans-gen	5.304	0.508
EPI-Trans-best	1.004	0.508

### Comparing the performance of the EPI-Trans model with state-of-the-art models

For fair comparison, we employed the same evaluation strategy used in state-of-the-art methods. We utilized the same TargetFinder EPI datasets that were used by previous state-of-the-art methods. In addition, we adopted the same strategy to split the datasets into training and testing datasets (i.e. same spilled ratio and same random seed), employing the same data augmentation technique to balance the training data, and using the same metrics (AUROC and AUPR) to evaluate the performance.

The performance of the best model “EPI-Trans-best” is compared with other cutting-edge techniques such as EPI-Mind, SPEID, PEP-WORD, EPIANN, SIMCNN, and EPI-DLMH. The results of these models are directly obtained from Yu Ni et al.’s work [37]. The comparison results are shown in Tables 8 and 9 in terms of AUROC and AUPR, respectively. In terms of AUROC, our proposed EPI-Trans-best model outperforms other models in three cell lines, including HeLa-S3, HUVEC, and K562, achieving impressive performance scores of 0.964, 0.952, and 0.956, respectively. Furthermore, the EPI-Trans-best model improves the performance of the HeLa-S3 cell line by 0.3%, 4.1%, 12.1%, 4%, 1.5%, and 1.2% over the performance of EPI-Mind, SPEID, PEP-WORD, EPIANN, SIMCNN, and EPI-DLMH, respectively.

Similarly, the model improved the performance of the HUVEC cell line by 0.7%, 4.8%, 10.7%, 3.4%, 1.9%, and 0.4% over the aforementioned models. Finally, the performance of the K562 cell line improved by 1%, 3.4%, 7.3%, 1.3%, 1.3%, and 0.1% respectively over the aforementioned models. For NHEK cell line, the proposed model is better than five out of six models which are SPEID, PEP-WORD, EPIANN, SIMCNN, and EPI-DLMH, and it achieved an increment of 3.3%, 6.6%, 2.4%, 2.1%, and 0.6% respectively, but it is worse than EPI-Mind only by 0.4%. For GM12878 cell line, it is better than the four models SPEID, PEP-WORD, EPIANN, and SIMCNN by 3%, 10.4%, 2.7%, and 0.5% respectively, and it is worse than the two EPI-Mind and EPI-DLMH models by 0.5% and 0.3% respectively. Finally, for IMR90 cell line is better than the three EPI-Mind, SPEID, and PEP-WORD models by 1.9%, 2.6%, and 4.3% respectively, but it achieved less performance than the other three EPIANN, SIMCNN, and EPI-DLMH models and the performance decreases by 0.4%, 1%, and 0.7% respectively.

On the other hand, the results showing the AUPR performance of the proposed EPI-Trans-best model and state-of-the-art methods for each cell line can be found in Table 9. The proposed model achieved the highest AUPR performance for HeLa-S3 cell line, with a score of 0.857 which is better than EPI-Mind, SPEID, PEP-WORD, EPIANN, SIMCNN, and EPI-DLMH by 1.4%, 6%, 5.4%, 15.5%, 12%, and 3.3%, respectively. While for NHEK, it achieved superior performance over the five SPEID, PEP-WORD, EPIANN, SIMCNN, and EPI-DLMH models, and the performance increased by 4.9%, 2.1%, 4%, 1.9%, and 0.8% respectively, but it is worse than EPI-Mind by 0.2%. Also for HUVEC cell line, the performance is better than the five EPI-Mind, SPEID, EPIANN, SIMCNN, and EPI-DLMH models by 1.4%, 20.1%, 10.8%, 8.4%, and 0.4% respectively, and it less than PEP-WORD model by 3.6% only. For GM12878, it outperforms SPEID, EPIANN, and SIMCNN models by 0.5%, 5.5%, and 7.2% respectively, and its performance is worse than EPI-Mind, PEP-WORD, and EPI-DLMH by 1.8%, 2.9%, and 4.1% respectively. The performance of the IMR90 cell line is better than the SPEID and SIMCNN models by 2.6% and 2.1% respectively, but it is worse than EPI-Mind, PEP-WORD, EPIANN, and EPI-DLMH by 1.1%, 11%, 1.2%, and 6% respectively.

Finally, the AUPR performance of the proposed EPI-Trans-best model for K562 cell line is better than EPIANN and SIMCNN models by 8.5% and 7.9% respectively, but its performance is less than EPI-Mind, SPEID, PEP-WORD, and EPI-DLMH by 0.2%, 1.3%, 7.8%, and 6.8% respectively. Overall, the average AUROC of EPI-Trans-best over the six cell lines is 95.7% which is better than the average AUROC of all other models, and the average AUPR is 79.6% which is better than SPEID, EPIANN, and SIMCNN models. So our EPI-Trans-best model outperforms most of the models in terms of AUROC and AUPR.

**Table 8:** Comparison between EPI-Trans-best model and other models in terms of AUROC

Model/cell lines	GM12878	HeLa-S3	HUVEC	IMR90	K562	NHEK	AVG
EPI-Trans-best	0.946	**0.964**	**0.952**	0.941	**0.956**	0.983	**0.957**
EPI-Mind-best	**0.951**	0.961	0.945	0.922	0.946	**0.987**	0.952
SPEID	0.916	0.923	0.904	0.915	0.922	0.950	0.922
PEP-WORD	0.842	0.843	0.845	0.898	0.883	0.917	0.871
EPIANN	0.919	0.924	0.918	**0.945**	0.943	0.959	0.935
SIMCNN	0.941	0.949	0.933	**0.951**	0.943	0.962	0.947
EPI-DLMH	**0.949**	0.952	0.948	**0.948**	0.955	0.977	0.955

The best performance in each cell line is given in boldface

**Table 9:** Comparison between EPI-Trans-best model and other models in terms of AUPR

Model/cell lines	GM12878	HeLa-S3	HUVEC	IMR90	K562	NHEK	AVG
EPI-Trans-best	0.778	**0.857**	0.724	0.758	0.758	0.901	0.796
EPI-Mind-best	**0.796**	0.843	0.710	**0.769**	0.756	**0.903**	0.796
SPEID	0.773	0.797	0.523	0.732	**0.771**	0.852	0.741
PEP-WORD	**0.807**	0.803	**0.760**	**0.868**	**0.836**	0.880	**0.826**
EPIANN	0.723	0.702	0.616	**0.770**	0.673	0.861	0.724
SIMCNN	0.706	0.737	0.640	0.737	0.679	0.882	0.730
EPI-DLMH	**0.819**	0.824	0.720	**0.818**	**0.826**	0.893	0.817

The best performance in each cell line is given in boldface

## Conclusion

This study introduces a novel deep-learning model that incorporates CNN and transformer mechanism. Initially, the proposed model employs the dna2vec embedding technique to convert tokens of enhancer/promoter sequences into vectors. Subsequently, a 2-layer CNN network extracts local features. Finally, a transformer processes the merged features of the enhancer and promoter as input. The inclusion of a transformer mechanism facilitates the extraction of features that effectively capture the extensive interconnections between enhancer and promoter sequences, thus enhancing the accuracy of predicting their relationship. Consequently, the model exhibits superior performance compared to other cutting-edge approaches across the majority of cell lines. Additionally, a generic model is proposed, capable of predicting enhancer–promoter interactions (EPIs) for any cell line used in training. The model’s performance is further enhanced by fine-tuning parameters through training on specific cell-line datasets. This enables the model to capture the unique features of the specific cell line, in addition to common features shared among all cell lines.

### Supplementary Information

**Additional file 1.** Detailed performance results comparing various optimizers, learning rates, batch sizes, and epoch numbers in terms of AUROC and AUPR.

## Data Availability

The data and the code are available at https://github.com/FMoonlightS/EPI-Trans/.

## Abbreviations

EPI: Enhancer–promoter interaction
GWAS: Genome-wide association studies
AUROC: Area under receiver operating characteristic curve
AUPR: Area under precision–recall curve
GRU: Gated recurrent unit
CNN: Convolutional neural network
LSTM: Long short-term memory
RNN: Recurrent neural network
Adam: Adaptive moment estimation
Nadam: Nesterov-accelerated adaptive moment estimation optimizer
