# Supplementary material for: EPI-Trans: an effective transformer-based deep learning model for enhancer promoter interaction prediction
Source: BMC Bioinformatics. 2024 Jun 18;25:216. doi: 10.1186/s12859-024-05784-9 (PMC11184834; doi:10.1186/s12859-024-05784-9)
Supplement: Supplementary file 1 — Additional file 1. Detailed performance results comparing various optimizers, learning rates, batch sizes, and epoch numbers in terms of AUROC and AUPR. [file 12859_2024_5784_MOESM1_ESM.pdf]

Supplementary Materials
EPI-Trans: An Effective Transformer-based Deep Learning Model for Enhancer Promoter Interaction Prediction

Table S 1: The performance of EPI-Trans-specific model due to using different optimizers in terms of AUROC on six different cell lines

Optimizer/ cell lines	GM12878	HeLa-S3	HUVEC	IMR90	K562	NHEK	AVG
Adam	**0.928**	0.959	0.933	0.902	0.920	0.979	0.937
SGD	0.912	0.957	0.925	0.885	0.932	**0.981**	0.932
RMSprop	0.922	**0.960**	0.922	0.894	0.927	0.975	0.933
Adamax	0.501	0.500	0.500	0.500	0.500	0.500	0.500
**Nadam**	0.927	0.952	**0.934**	**0.909**	**0.935**	0.975	**0.939**

Note: The best performance in each cell line, the best average, and the final optimizer that is used in our experiments are given in boldface

Table S 2: The performance of EPI-Trans-specific model due to using different optimizers in terms of AUPR on six different cell lines

Optimizer/ cell lines	GM12878	HeLa-S3	HUVEC	IMR90	K562	NHEK	AVG
Adam	0.743	0.848	0.705	0.713	**0.764**	0.896	0.778
SGD	0.692	0.768	0.629	0.636	0.712	0.869	0.718
RMSprop	0.777	0.838	0.689	0.716	0.735	0.892	0.774
Adamax	0.048	0.048	0.048	0.048	0.048	0.048	0.048
**Nadam**	**0.788**	**0.855**	**0.718**	**0.721**	0.755	**0.902**	**0.790**

Note: The best performance in each cell line, the best average, and the final optimizer that is used in our experiments are given in boldface

Table S 3: The performance of EPI-Trans-specific model due to using different learning rates of Nadam optimizer in terms of AUROC on six different cell lines

Learning Rate/ cell lines	GM12878	HeLa-S3	HUVEC	IMR90	K562	NHEK	AVG
0.01	0.892	0.932	0.893	0.872	0.908	0.960	0.910
**0.001**	**0.927**	0.952	**0.934**	**0.909**	**0.935**	0.975	**0.939**
0.0001	0.901	**0.954**	0.915	0.885	0.920	**0.977**	0.925

Note: The best performance in each cell line, the best average, and the final value of the learning rate that is used in our experiments are given in boldface

Table S 4: The performance of EPI-Trans-specific model due to using different learning rates of Nadam optimizer in terms of AUPR on six different cell lines

Learning Rate/ cell lines	GM12878	HeLa-S3	HUVEC	IMR90	K562	NHEK	AVG
0.01	0.432	0.518	0.445	0.512	0.354	0.727	0.498
**0.001**	**0.788**	**0.855**	**0.718**	**0.721**	**0.755**	**0.902**	**0.790**
0.0001	0.700	0.791	0.586	0.645	0.727	0.849	0.716

Note: The best performance in each cell line, the best average, and the final value of the learning rate that is used in our experiments are given in boldface

Table S 5: The performance of EPI-Trans-specific model due to using different batch sizes, and using Nadam optimizer with learning rate 0.001 in terms of AUROC on six different cell lines

Batch Size/ cell lines	GM12878	HeLa-S3	HUVEC	IMR90	K562	NHEK	AVG
16	0.921	**0.964**	0.931	0.878	0.930	0.980	0.934
32	0.921	0.956	0.919	**0.911**	0.931	0.977	0.936
**64**	0.928	0.960	**0.943**	0.888	0.943	**0.982**	**0.941**
128	**0.930**	**0.964**	0.926	0.898	0.928	0.976	0.937
256	0.910	0.958	0.927	0.898	**0.945**	**0.982**	0.937

Note: The best performance in each cell line, the best average, and the final value of the batch size that is used in our experiments are given in boldface

Table S 6: The performance of EPI-Trans-specific model due to using different batch sizes, and using Nadam optimizer with learning rate 0.001 in terms of AUPR on six different cell lines

Batch Size/ cell lines	GM12878	HeLa-S3	HUVEC	IMR90	K562	NHEK	AVG
16	0.726	0.712	0.643	0.670	0.759	0.882	0.732
32	0.770	0.859	0.669	0.730	0.771	0.889	0.781
**64**	0.779	0.849	**0.713**	**0.742**	**0.783**	**0.919**	**0.797**
128	**0.790**	0.859	0.691	0.720	0.753	0.914	0.788
256	0.707	**0.865**	0.650	0.683	0.743	0.908	0.759

Note: The best performance in each cell line, the best average, and the final value of the batch size that is used in our experiments are given in boldface

Table S 7: The performance of EPI-Trans-specific model due to using different values for epochs, using Nadam optimizer with learning rate 0.001, and batch size 64 in terms of AUROC on six different cell lines

Epochs/cell lines	GM12878	HeLa-S3	HUVEC	IMR90	K562	NHEK	AVG
15	0.928	0.960	**0.943**	0.888	**0.943**	0.982	**0.941**
**20**	**0.933**	**0.963**	0.939	0.898	0.931	**0.984**	**0.941**
25	0.929	**0.963**	0.926	0.893	0.934	0.980	0.938
30	0.929	**0.963**	0.923	**0.911**	0.935	0.981	0.940

Note: The best performance in each cell line, the best average, and the final value of the number of epochs that is used in our experiments are given in boldface

Table S 8: The performance of EPI-Trans-specific model due to using different values for epochs, using Nadam optimizer with learning rate 0.001, and batch size 64 in terms of AUPR on six different cell lines

Epochs/cell lines	GM12878	HeLa-S3	HUVEC	IMR90	K562	NHEK	AVG
15	0.779	0.849	0.713	0.742	**0.783**	0.919	0.797
**20**	0.795	0.854	**0.736**	0.733	**0.783**	**0.922**	**0.804**
25	0.788	0.852	0.730	0.746	0.774	0.918	0.801
30	**0.800**	**0.861**	0.711	**0.747**	0.782	0.919	0.803

Note: The best performance in each cell line, the best average, and the final value of the number of epochs that is used in our experiments are given in boldface

Table S 9: The performance of EPI-Trans-general model due to using different values for epochs, using Nadam optimizer with learning rate 0.001, and batch size 64 in terms of AUROC on six different cell lines

Epochs/cell lines	GM12878	HeLa-S3	HUVEC	IMR90	K562	NHEK	AVG
20	0.938	0.955	0.941	0.932	0.940	0.967	0.946
**25**	**0.944**	**0.963**	**0.944**	**0.933**	0.942	**0.975**	**0.950**
30	0.940	0.958	0.938	0.929	**0.943**	0.971	0.947

Note: The best performance in each cell line, the best average, and the final value of the number of epochs that is used in our experiments are given in boldface

Table S 10: The performance of EPI-Trans-general model due to using different values for epochs, using Nadam optimizer with learning rate 0.001, and batch size 64 in terms of AUPR on six different cell lines

Epochs/cell lines	GM12878	HeLa-S3	HUVEC	IMR90	K562	NHEK	AVG
20	**0.668**	0.715	0.564	0.607	**0.666**	0.716	0.656
**25**	0.643	**0.749**	0.584	**0.611**	0.658	0.723	**0.661**
30	0.629	0.728	**0.585**	0.606	0.645	**0.731**	0.654

Note: The best performance in each cell line, the best average, and the final value of the number of epochs that is used in our experiments are given in boldface

Table S 11: The performance of EPI-Trans-best model due to using different values for epochs, using Nadam optimizer with learning rate 0.001, and batch size 64 in terms of AUROC on six different cell lines

Epochs/cell lines	GM12878	HeLa-S3	HUVEC	IMR90	K562	NHEK	AVG
20	0.945	0.963	**0.954**	0.934	0.952	**0.985**	0.956
25	**0.947**	**0.969**	0.942	0.931	0.941	0.980	0.952
**30**	0.946	0.964	0.952	**0.941**	**0.956**	0.983	**0.957**

Note: The best performance in each cell line, the best average, and the final value of the number of epochs that is used in our experiments are given in boldface

Table S 12: The performance of EPI-Trans-best model due to using different values for epochs, using Nadam optimizer with learning rate 0.001, and batch size 64 in terms of AUPR on six different cell lines

Epochs/cell lines	GM12878	HeLa-S3	HUVEC	IMR90	K562	NHEK	AVG
20	0.763	0.838	**0.732**	0.758	**0.770**	0.894	0.793
25	0.742	0.846	0.727	**0.760**	0.731	0.876	0.780
**30**	**0.778**	**0.857**	0.724	0.758	0.758	**0.901**	**0.796**

Note: The best performance in each cell line, the best average, and the final value of the number of epochs that is used in our experiments are given in boldface

Table S 13: The training time for all samples in minutes of the EPI-Trans models for the six cell lines

Model/Cell line	GM12878	HeLa-S3	HUVEC	IMR90	K562	NHEK	AVG
EPI-Trans-spec	49.621	54.620	53.646	42.424	59.760	45.525	50.932
EPI-Trans-gen	-	-	-	-	-	-	318.220
EPI-Trans-best	75.397	65.100	56.247	45.364	73.012	46.254	60.229

Table S 14: The test time per a sam in milliseconds of the EPI-Trans models for the six cell lines

Model/Cell line	GM12878	HeLa-S3	HUVEC	IMR90	K562	NHEK	AVG
EPI-Trans-spec	0.868	0.427	0.453	0.424	0.436	0.415	0.504
EPI-Trans-gen	0.878	0.425	0.472	0.417	0.445	0.409	0.508
EPI-Trans-best	0.842	0.436	0.471	0.431	0.446	0.422	0.508
